# Development of sensitized solar cells by photosynthetic pigments

**DOI:** 10.1186/1753-6561-8-S4-P242

**Published:** 2014-10-01

**Authors:** Iara Mendes Maciel, Maria José Otero Martino, Raúl Barba Tamaro, Luis José Andrés Menendez

**Affiliations:** 1 Universidade Federal de Goiás, Goiânia, Goiás, Brazil; 2 Universidad de Oviedo, Oviedo, Asturias, Spain; 3 ITMA Materials Technology, Avilés, Asturias, Spain

## Background

Grätzel solar cell, also known as dye-sensitized solar cell (DSSC), is a dye-sensitized nanocrystalline photovoltaic cell of TiO_2 _(titanium dioxide). It has a surface of nanoporous TiO_2_, a semiconductor of a wide bandgap. The basic principle is to transform light energy into electrical energy. When a dye molecule absorbs light, an electron is replaced by an excited state, and this dye can jump to the conduction band of TiO_2_. In the composite electrode, the electron diffuses from this TiO_2 _to the conductive glass. From there, the electron is taken to the electrode through a conductor. Similar to this process, the plant cell transforms the light into energy using photosynthetic pigments for absorption and many other mechanisms for the transfer and storage of energy, by a process known as photosynthesis. Observing and analyzing these methods, our objective was to use some photosynthetic pigments found in plants in Grätzel solar cells to evaluate the potential use of these pigments in the production of solar panels.

## Methods

The extraction of pigments was performed of 4 algae: *Codium tomentosum *(CT), *Ulva lactuca *(UL), *Ahnfeltia plicata *(AP), *Pterosiphonia complanata *(PC); 2 fruits: *Rubus ulmifolius *(ANM)*, Prunus spinosa *(AN); and spinach leaves: *Spinacia oleracea *(CL), using 100% acetone in the case of algae and leaves (extraction of chlorophylls, carotenoids and xanthophylls) and 0.1-1%HCl (pH<2) in ethanol in the case of the fruits (extraction of anthocyanins).

A TLC was performed with a filter paper using a mixture of hexane:acetone (7:3), an absorbance reading of the extracts for the separation and a verification of pigments found in each sample.

Subsequently, DSSCs were constructed. Was added a TiO_2 _(liq) layer in the conductive glass, heated at 450°C to attach to the glass layer. Then, submerged in the pigments solutions for 24 hours. After that, another conductive glass scratched with graffiti was arranged and closed up with tweezers. A few drops of solution iodine/iodide were added. The DSSCs were connected in a multimeter and were putted in a solar simulator, with the objective of measuring the voltage when stimulated and unstimulated by light in different time periods.

## Results and conclusions

Analyzing the samples voltage in the presence and absence of light, it can be concluded that the voltage produced in the absence of light is insignificant when compared to the voltage at light.

Of all samples measured, the ones that showed the best results in relation of the voltage and stability over a certain period of time were those with a higher proportion of chlorophyll: UL and AP. CL also showed elevated values, though quite ranged over the days, which may be due the degradation of the pigment. PC and AN had high stability but very low voltage. ANM showed irregular and low results.

Continuing with the samples for several days, it might be concluded that the cells with pigments of AP and UL remain in operation longer than expected, and UL, moreover, maintains stability. Thus, the use of photosynthetic pigments for the solar cells production from this study is feasible.

